# Mucociliary transport deficiency and disease progression in Syrian hamsters with SARS-CoV-2 infection

**DOI:** 10.1172/jci.insight.163962

**Published:** 2023-01-10

**Authors:** Qian Li, Kadambari Vijaykumar, Scott E. Phillips, Shah S. Hussain, Nha V. Huynh, Courtney M. Fernandez-Petty, Jacelyn E. Peabody Lever, Jeremy B. Foote, Janna Ren, Javier Campos-Gómez, Farah Abou Daya, Nathaniel W. Hubbs, Harrison Kim, Ezinwanne Onuoha, Evan R. Boitet, Lianwu Fu, Hui Min Leung, Linhui Yu, Thomas W. Detchemendy, Levi T. Schaefers, Jennifer L. Tipper, Lloyd J. Edwards, Sixto M. Leal, Kevin S. Harrod, Guillermo J. Tearney, Steven M. Rowe

**Affiliations:** 1 Department of Medicine,; 2 Gregory Fleming James Cystic Fibrosis Research Center,; 3 Graduate Biomedical Sciences Program,; 4 Department of Microbiology,; 5 Department of Chemistry,; 6 Department of Radiology, and; 7 Department of Biomedical Engineering, University of Alabama at Birmingham, Birmingham, Alabama, USA.; 8 Wellman Center for Photomedicine, Massachusetts General Hospital, Harvard Medical School, Boston, Massachusetts, USA.; 9 Department of Anesthesiology and Perioperative Medicine,; 10 Department of Biostatistics,; 11 Department of Pediatrics,; 12 Department of Cell Developmental and Integrative Biology, University of Alabama at Birmingham, Birmingham, Alabama, USA.

**Keywords:** COVID-19, Respiration

## Abstract

Substantial clinical evidence supports the notion that ciliary function in the airways is important in COVID-19 pathogenesis. Although ciliary damage has been observed in both in vitro and in vivo models, the extent or nature of impairment of mucociliary transport (MCT) in in vivo models remains unknown. We hypothesize that SARS-CoV-2 infection results in MCT deficiency in the airways of golden Syrian hamsters that precedes pathological injury in lung parenchyma. Micro-optical coherence tomography was used to quantitate functional changes in the MCT apparatus. Both genomic and subgenomic viral RNA pathological and physiological changes were monitored in parallel. We show that SARS-CoV-2 infection caused a 67% decrease in MCT rate as early as 2 days postinfection (dpi) in hamsters, principally due to 79% diminished airway coverage of motile cilia. Correlating quantitation of physiological, virological, and pathological changes reveals steadily descending infection from the upper airways to lower airways to lung parenchyma within 7 dpi. Our results indicate that functional deficits of the MCT apparatus are a key aspect of COVID-19 pathogenesis, may extend viral retention, and could pose a risk factor for secondary infection. Clinically, monitoring abnormal ciliated cell function may indicate disease progression. Therapies directed toward the MCT apparatus deserve further investigation.

## Graphical abstract

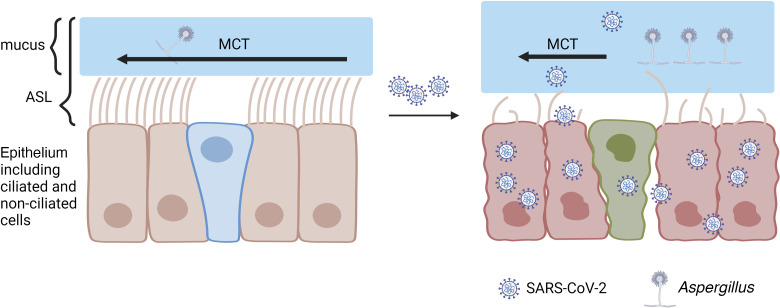

## Introduction

Coronavirus disease 2019 (COVID-19), caused by severe acute respiratory syndrome coronavirus 2 (SARS-CoV-2), is dominated by respiratory disease, including pneumonia (1–4). SARS-CoV-2 binds to its cellular receptor, angiotensin-converting enzyme 2 (ACE2), initiating cell entry and subsequent pathogenesis (5–9). The degree of ACE2 expression is thought to impart susceptibility to SARS-CoV-2 and thus the severity of the disease upon infection (10–13). Single-cell RNA sequencing (scRNA-seq) from healthy donors has demonstrated high expression of ACE2 in respiratory epithelial cells (14), indicating tropism of SARS-CoV-2 infection for the respiratory tract (15–17). Epithelial cells represent the first contact of respiratory viruses with the host, followed by pathological progression to the deep lung and other tissues as descending infection occurs. Thus, the functional consequences of epithelial cell infection in COVID-19 are crucial to understanding its pathogenesis.

Among respiratory epithelial cells, ACE2 expression dominates in ciliated cells (14), a cell type found throughout the conducting airways and crucial for maintaining mucociliary transport (MCT), a critical host defense mechanism. Excessive mucin production and hyperviscous mucus have been frequently found in bronchoalveolar lavage fluid (BALF) (18–20) and airways (21–23) of COVID-19 patients. Perhaps consequently, bacterial or fungal superinfection occurs in up to one-third of COVID-19 patients (24–27) and contributes to increased severity and mortality (25–28). In vitro studies using differentiated human tracheobronchial epithelial (HTBE) cells showed SARS-CoV-2 infection leads to shorter cilia and abolished ciliary beating in the center of cytopathic plaques, with additional morphologic changes to cilia in neighboring regions (29, 30). Impaired MCT was reported in HTBE cells measured by tracking polystyrene beads (31). These reports suggest that diminished MCT could contribute to descending viral infection and secondary infections, but presently remains unknown for the intact mucociliary clearance (MCC) apparatus (i.e., tissue-level analysis) from the perspective of an in vivo model of disease.

Syrian hamsters have been used to model respiratory infections by SARS-CoV, influenza virus, and adenovirus (32–35). ACE2 sequence homology at the SARS-CoV-2 receptor binding domain has shown hamsters to have the most similarity to humans, second only to nonhuman primates (36). Furthermore, hamsters with SARS-CoV-2 exhibit virological, physiological, and pathological endpoints relevant to human disease (37–40), being applied to various studies including mechanistic investigation (36, 41–45), vaccine (46–49), antibody (50, 51), and therapeutic development (52). Here, we follow the time courses of virological, physiological, and pathological changes in hamsters up to 7 days postinfection (dpi) with SARS-CoV-2, as well as changes in the MCT apparatus using micro-optical coherence tomography (μOCT), a powerful method to visualize and simultaneously quantitate multiple aspects of the functional microanatomy of airways in intact tissues (53).

## Results

### SARS-CoV-2 induces weight loss and lethargy in hamsters.

To assess the effects of SARS-CoV-2 infection on the MCT apparatus, we first established the model in golden Syrian hamsters, an animal model that recapitulates human COVID-19 (37–40). Under BSL-3 conditions and as shown in Supplemental Figure 1 (supplemental material available online with this article; https://doi.org/10.1172/jci.insight.163962DS1), following nasal inoculation of 3 × 10^5^ plaque-forming units (PFU) of SARS-CoV-2 in 100 μL of viral propagation media, hamsters exhibited progressive weight loss up to 10.2% ± 1.0% of body weight (BW), peaking at 6 dpi (*P* = 0.0052) as compared with the stable BW observed in mock controls evaluated by the same experimental procedures. This is consistent with previous hamster studies using various strains and titers of SARS-CoV-2 that demonstrated peak BW loss that ranged from 10%–20% and peaked between 5 and 7 dpi (36, 39–41, 43, 44, 46, 48, 54). We also observed moderate lethargy through 7 dpi in SARS-CoV-2–infected hamsters but not in mock controls, although other clinical signs such as changes in fur or posture, sneezing, coughing, diarrhea, and nasal or ocular discharge were not found. This clinical syndrome was also consistent with previous reports using other strains of SARS-CoV-2 in hamsters (36, 38).

### SARS-CoV-2 is prominently located in the respiratory tract.

**Figure 1:**
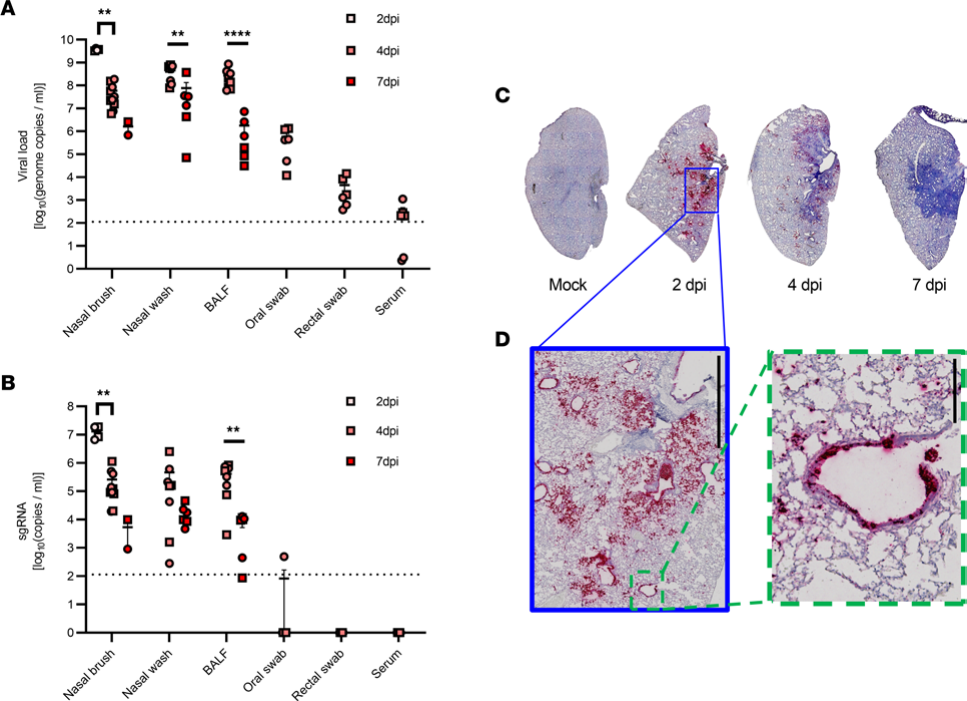
SARS-CoV-2 detection in hamsters through 7 dpi. Golden Syrian hamsters were inoculated intranasally with 3 × 10^5^ PFU of SARS-CoV-2 or vehicle (mock), and samples were collected at 0, 2, 4, and 7 dpi. Genomic (**A**) and subgenomic (**B**) viral titers were quantitated by qRT^2^-PCR versus standard curve for nasal brush (*n* = 4, 10, and 2 for 2, 4, and 7dpi, respectively), nasal wash (*n* = 8 and 6 for 4 and 7 dpi, respectively), bronchial alveolar lavage fluid (BALF, *n* = 8 and 6 for 4 and 7 dpi, respectively), oral swab (*n* = 6 for 4 dpi), rectal swab (*n* = 6 for 4 dpi), and serum (*n* = 6 for 4 dpi). Viral titers in all types of samples from mock were under the detection limit and are not shown. Subgenomic viral titers in serum, rectal swab, and most oral swabs were below quantitation limits by PCR, and are shown as 0. The dotted line indicates the limits of the PCR method. Error bars show the SEM. Squares represent males and circles females. Values in log_10_(copies/mL) were used for statistical analysis. For nasal brush, *P* < 0.0001 and *P* = 0.0161 for genomic and subgenomic titers, respectively, by 2-way ANOVA, with **P < 0.01 (shown by a bracket) by Tukey’s post hoc test. For nasal wash and BALF, **P < 0.01 and ****P < 0.0001 (shown by straight lines) by unpaired *t* test. Representative images of the whole left lobe slices (*n* = 2 per time point) with SARS-CoV-2 detection by RNAscope (**C**), with high-power view (**D**) of the airway (left, scale bar: 2 mm) and parenchymal lung (right, scale bar: 0.2 mm) at 2 dpi.

To evaluate viral infection, we detected SARS-CoV-2 in hamsters using complementary techniques. Figure 1A shows viral load in various samples at 2, 4, and 7 dpi quantified by quantitative real-time reverse transcriptase–polymerase chain reaction (qRT^2^-PCR). We detected high viral titers in the respiratory tract that exceeded 1 × 10^7^ genome copies/mL through 4 dpi, including nasal brush, nasal wash, and BALF specimens. Viral titers in these respiratory samples were notably greater than titers in either gastrointestinal tract (oral and rectal swabs were approximately 1 × 10^5^ and 1 × 10^3^ copies/mL, respectively) or circulation (i.e., serum, approximately 1 × 10^2^ copies/mL). Although differences in sample collection methods or sample volume may have contributed to the differences observed, viral titers in the respiratory tract exceeded other tissues by at least 3 orders of magnitude. This indicated important differences among these tissues that make the respiratory tract the main target of viral invasion, which is consistent with the tissue specificity reported by Chan et al. (36). Higher viral load in BALF than those in oral and rectal swabs has also been found in COVID-19 patients (9).

We also performed an analysis using primers to detect subgenomic (sg) SARS-CoV-2 RNA by qRT^2^-PCR to confirm SARS-CoV-2 replication. Along with the analysis of genomic copies, the respiratory tract was the dominant site of viral replication (Figure 1B). At 4 dpi, sgRNA levels exceeded 1 × 10^5^ copies/mL in the nasal brush, nasal wash, and BALF specimens, whereas in oral, rectal swabs, and sera, those were below quantitation limits.

The viral load decreased with time by examining differences in viral titers of the respiratory tract within the same sample type (Figure 1A). In nasal brush specimens, the viral RNA copy number at 2, 4, and 7 dpi decreased significantly from 9.6 ± 0.0 to 7.5 ± 0.2 and then 6.1 ± 0.3 log_10_(copies/mL), respectively (*P* < 0.0001). For nasal wash and BALF, similar findings were shown at 4 and 7 dpi, with a reduction of 8.5 ± 0.1 to 7.0 ± 0.5 log_10_(copies/mL) for nasal wash (*P* = 0.0077), and 8.3 ± 0.1 to 5.6 ± 0.4 log_10_(copies/mL) for BALF (*P* < 0.0001). Levels of sgRNA (Figure 1B) detected within the same sample type in the respiratory tract also diminished with time (7.1 ± 0.1 vs. 5.1 ± 0.2 vs. 3.5 ± 0.5 log_10_(copies/mL) at 2, 4, and 7 dpi for nasal brush, *P* = 0.0161; 5.3 ± 0.3 vs. 3.5 ± 0.4 log_10_(copies/mL) for BALF at 4 and 7 dpi, *P* = 0.0021). High viral loads at the onset of infection that diminished over time are consistent with previous reports (36, 40–42, 48).

Next, we examined SARS-CoV-2 RNA distribution in hamster lungs by in situ hybridization with RNAscope. Each viral RNA transcript was hybridized and then the detection signal was visualized as a point of amplification. Figure 1, C and D show that SARS-CoV-2 in the lung exhibited an airway-centric pattern. Large and medium airways exhibited the most prominent viral RNA detection, whereas parenchymal distribution was patchy and heterogeneous (Figure 1D). This pattern evolved, with peak detection at 2 dpi that remained prominent and airway-centric at 4 dpi, but was absent by 7 dpi (Figure 1C). Together with genomic and viral sgRNA found in respiratory samples by qRT^2^-PCR, our results demonstrate prominent viral RNA presence in the respiratory airways that is most evident in the large and medium airways and progresses to the distal lung thereafter. The data further show clearance of SARS-CoV-2 at 7 dpi, which is consistent with the recovery and BW gain after 6 or 7 dpi (36, 39–41, 44, 46, 48). These findings indicate respiratory epithelial cells as the main target of initial viral infection.

### SARS-CoV-2 infection causes lung injury in hamsters.

**Figure 2:**
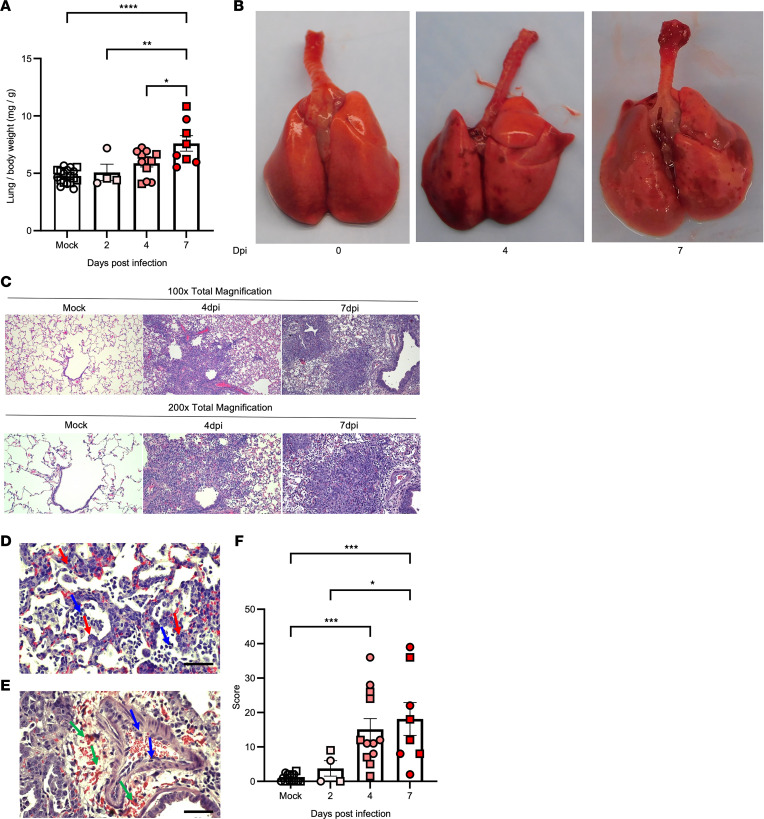
Lung injury after SARS-CoV-2 infection in hamsters. Golden Syrian hamsters were inoculated as in Figure 1, and lung damage was assessed by lung to body weight ratio (**A**, *n* = 18, 2, 12, and 8 for mock, 2, 4, and 7 dpi, respectively), gross pathology (**B**, representative of *n* = 18, 12, and 10 for mock, 4, and 7 dpi, respectively), and histopathological analysis of the left lung, including representative H&E images (**C**–**E**, representatives of *n* = 18, 12, and 8 for mock, 4, and 7 dpi, respectively) and quantitation by a blinded pathologist (**F**, *n* = 16, 4, 12, and 8 for mock, 2, 4, and 7 dpi, respectively). (**C**) Total magnification ×100 (top) and ×200 (bottom) of lungs from mock, 4 dpi, and 7 dpi displaying progression to interstitial pneumonia. (**D**) Total magnification ×400 of alveolus and alveolar interstitium displaying type II pneumocyte hyperplasia (red arrows) and mononuclear infiltrate (blue arrows) and (**E**) a small-caliber artery displaying perivascular edema, hemorrhage (green arrows), and intimal arteritis (blue arrows). Scale bar: 50 μm. Error bars indicate SEM. Squares indicate males and circles females. (**A** and **F**) *P* < 0.0001 by 1-way ANOVA. *P < 0.05; **P < 0.01; ***P < 0.001; ****P < 0.0001 by Tukey’s post hoc test.

The major focus of severe COVID-19 has been the injury to the distal lung parenchyma; accordingly, we evaluated lesions in the lung to characterize the pathology induced by SARS-CoV-2 infection in hamsters. As SARS-CoV-2 was gradually depleted, the lung damage caused by initial viral invasion progressed with time and increased in severity through 7 dpi, in concert with changes in viral replication (Figure 2). The lung to BW ratio (L/BW) (Figure 2A) steadily increased by 62% from 4.7 ± 0.1 in mock to 7.6 ± 0.7 mg/g at 7 dpi (*P* < 0.0001), suggesting progressive development of edema and cell infiltration. Multifocal consolidation and hyperemia were apparent as early as 4 dpi and were greatest at 7 dpi on gross lung inspection (Figure 2B). The hematoxylin and eosin–stained (H&E-stained) lungs of SARS-CoV-2–infected hamsters revealed a primarily patchy and multifocal interstitial pneumonia, with lesion severity most prominent between 4 and 7 dpi (Figure 2C). The histopathologic characterization of lesion spectra revealed (a) type II pneumocyte hyperplasia (Figure 2D), (b) interstitial and alveolar mixed mononuclear cell infiltrate (Figures 2, D and E), and (c) perivascular edema and hemorrhage (Figure 2E). Using scoring criteria developed by Gruber et al. (55), the quantitation of the lung histopathology illustrated the progression of lung lesions in SARS-CoV-2–infected hamsters (Figure 2F; 1 ± 0, 4 ± 2, 15 ± 3, and 18 ± 5 for mock, 2, 4, and 7 dpi, respectively), consistent with changes in the L/BW ratio, gross pathology, and a previous report (54). Importantly, these lesions have also been observed in COVID-19 patients (40, 56, 57).

### SARS-CoV-2 causes defective MCT.

**Figure 3:**
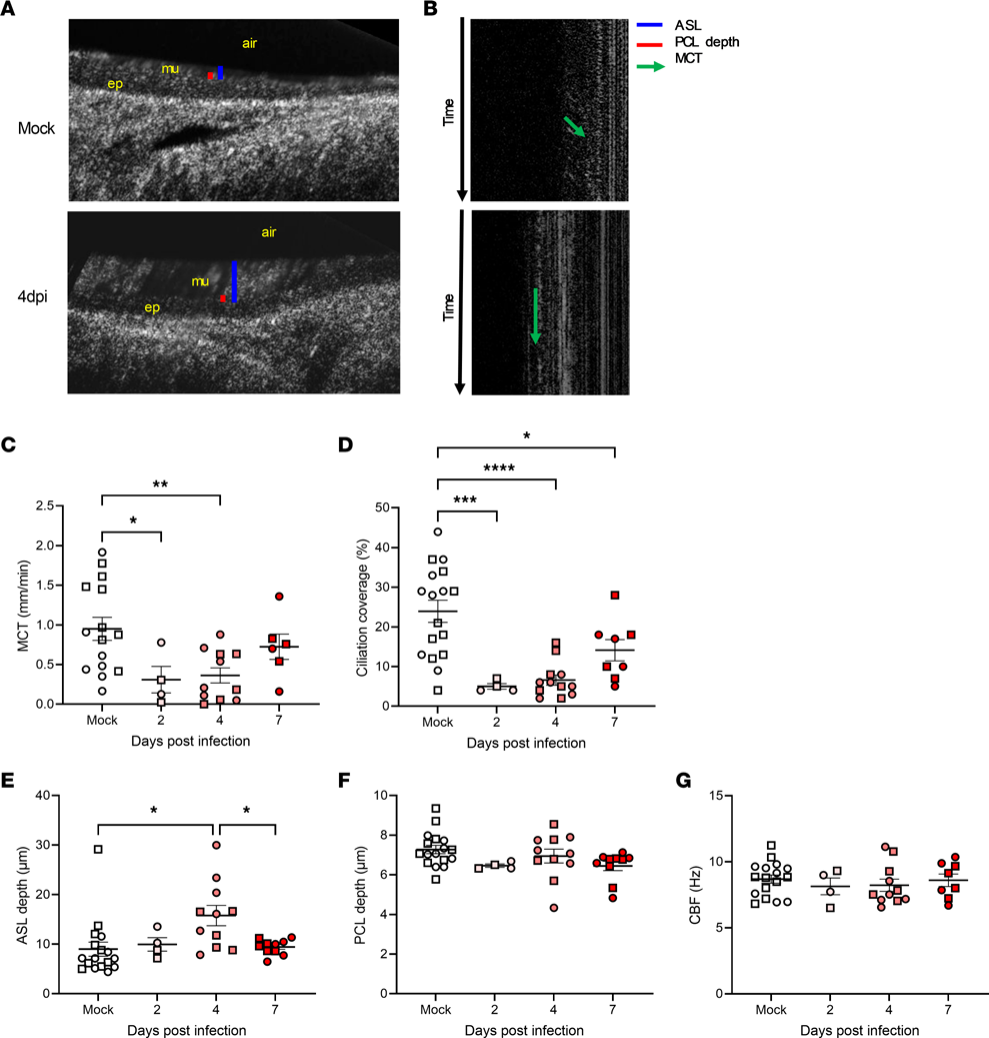
Mucociliary dysfunction in hamster airways after SARS-CoV-2 infection. Golden Syrian hamsters were inoculated as in Figure 1. Following excision, tracheas were imaged by micro-optical coherence tomography (μOCT, **A**–**G**). Representative μOCT images (*n* = 17 and 12 for mock and 4 dpi, respectively) from mock and 4 dpi (**A**) and M-mode projections of μOCT videos (**B**). ep, epithelium; mu, mucus. Mucociliary transport (MCT) rate (**C**, *n* = 15, 4, 11, and 6 for mock, 2, 4, and 7 dpi, respectively), degree of active ciliation coverage (**D**, *n* = 17, 4, 12, and 8 for mock, 2, 4, and 7 dpi, respectively), depths of airway surface liquid (ASL) (**E**, *n* = 17, 4, 11, and 10 for mock, 2, 4, and 7 dpi, respectively) and periciliary layer (PCL) (**F**, *n* = 17, 4, 11, and 10 for mock, 2, 4, and 7 dpi, respectively), and ciliary beat frequency (CBF) (**G**, *n* = 17, 4, 11, and 8 for mock, 2, 4, and 7 dpi, respectively) are quantitated. Error bars indicate SEM. Squares indicate males and circles females. *P* = 0.0099, *P* < 0.0001, *P* = 0.0117, *P* = 0.1106, *P* = 0.7808 by 1-way ANOVA for **C**–**H**, respectively. *P < 0.05; **P < 0.01; ***P < 0.001; ****P < 0.0001 by Tukey’s post hoc test.

Having established that moderately severe SARS-CoV-2 respiratory infection in hamsters exhibits prominent airway involvement, as with human COVID-19, we examined the effects of SARS-CoV-2 infection on the functional microanatomy of tracheas. μOCT allows the visualization and simultaneous quantitation of 5 microanatomic parameters that characterize the MCC apparatus: airway surface liquid (ASL) depth and periciliary liquid (PCL) depth, which are tightly associated with ciliary height (CH); cilia beat frequency (CBF); the area of active ciliary beating, which is termed motile ciliation coverage (CC); and ultimately MCT rate. The representative images for mock and at 4 dpi (Figure 3A) with corresponding M-mode resliced images to measure the MCT rate of native particles within mucus (Figure 3B) are shown (also see real-time recordings in Supplemental Videos 1 and 2). Multiple functional deficits were readily apparent, including diminished ciliary beating and reduced MCT rate upon SARS-CoV-2 infection. Particle transport quantitation demonstrated a diminished MCT rate, from 0.95 ± 0.14 mm/min in mock to 0.31 ± 0.17 mm/min (*P* = 0.0479) and 0.36 ± 0.10 mm/min (*P* = 0.0076) at 2 and 4 dpi, respectively, whereas MCT had partially recovered by 7 dpi (0.72 ± 0.16 mm/min) (Figure 3C). This 67% and 62% decrease (at 2 and 4 dpi, respectively) in MCT as infection peaked within the epithelium was closely associated with diminished motile CC, which was reduced from 23.9% ± 2.8% in mock to 5.0% ± 0.7% (*P* = 0.0010), 6.6% ± 1.3% (*P* < 0.0001), and 14.1% ± 2.7% (*P* = 0.0324) at 2, 4, and 7 dpi, respectively, representing 21%–28% of the normal level at peak viral replication/infection that partially recovered to 59% of mock by 7 dpi (Figure 3D). With greater ciliation compared with infected tracheas, there are more regions of interest (ROIs) that have MCT and CC for mock tracheas, which could contribute to the variability of MCT and CC values of mock tracheas. The baseline CC value first measured here seems reasonable, considering that half of cells in the conducting airways of hamsters express cilia based on a pathological study (58). Another notable feature (Figure 3E) included an increase in ASL depth at 4 dpi (from 9.0 ± 1.4 in mock to 15.8 ± 2.0 μm, *P* = 0.0100), likely reflecting the accumulation of mucus due to delayed MCT rate, that resolved as CC and MCT began to recover at 7 dpi (9.4 ± 0.5 μm vs. 4 dpi, *P* = 0.0420). PCL depth (Figure 3F) exhibited no statistically significant changes, although a trend was evident, and measurements may have been limited by reduced resolution under BSL-3 containment. Residual cilia with detectable motion had no meaningful changes in mean CBF (Figure 3G). The baseline CBF (8.7 ± 0.3 Hz) in hamster trachea is comparable to 13.7 ± 3.1 Hz previously measured by a photoelectric system equipped with a fiber-optic probe (59).

To quantitatively ascertain contributors to the MCT defect due to SARS-CoV-2 infection, we performed regression analysis. The regression analysis of MCC parameters by animals demonstrated that SARS-CoV-2 infection (β = –0.495, *P* = 0.003) and CC (β = 0.018, *P* = 0.019) were each associated with delayed MCT (Table 1). To further explore the relationship between reduced MCT rate and SARS-CoV-2 infection, we conducted a linear mixed-model analysis. Our initial model contained COVID status, dpi, and ROI (500-mm specific field of view) as fixed effects, as well as random animal-specific intercept. In this model, dpi and ROI were not statistically significant, but COVID status was statistically significant (*P* = 0.0287). Consequently, ROI was removed as a fixed effect and replaced with sex and cohort as fixed effects with random animal-specific intercept. This model, including experimental cohort, dpi, sex, and infection status as fixed effects, confirmed that SARS-CoV-2 infection was the only variable that had significant effects on MCT rate (*P* = 0.0389).

### SARS-CoV-2 causes tracheal and ciliary injury in hamsters.

**Figure 4:**
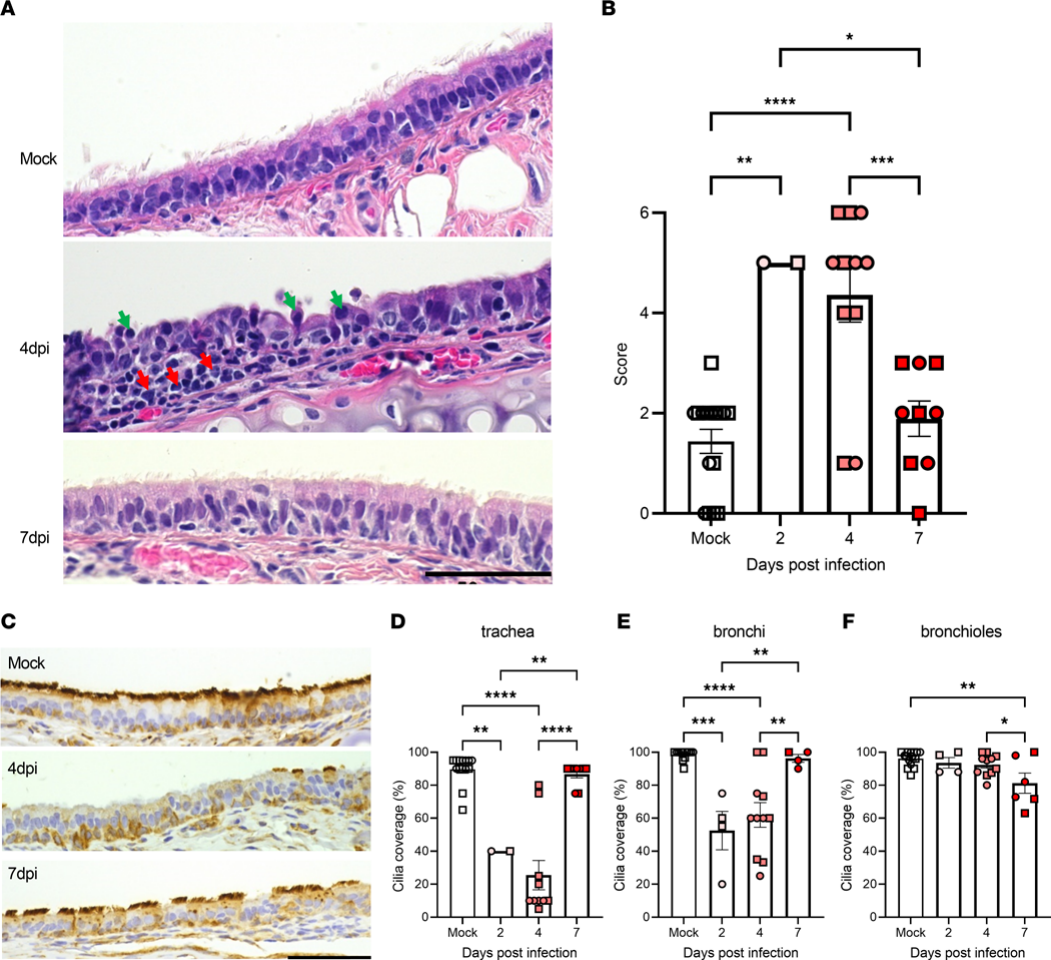
Tracheal injury after SARS-CoV-2 infection in hamsters. After μOCT imaging in Figure 3, hamster tracheas were processed for histopathological analysis. Representative H&E images (**A**) and lesion quantitation by a blinded pathologist (**B**) are shown (*n* = 16, 2, 11, and 9 for mock, 2, 4, and 7 dpi, respectively). Red arrows indicate mononuclear inflammatory cells that expanded the submucosa and infiltrated the epithelial mucosal layer. Green arrows indicate apoptotic, desquamated epithelial cells that lost the attachment to adjacent epithelia. Unstained slides of tracheas and lungs from Figure 2 were labeled for α-tubulin by IHC to specifically focus on ciliary injury. Representative images are shown of trachea (**C**), and quantitation of cilia coverage along the apical surface of the tracheal (**D**, *n* = 14, 2, 10, and 9 for mock, 2, 4, and 7 dpi, respectively), bronchi (**E**, *n* = 13, 4, 11, and 4 for mock, 2, 4, and 7 dpi, respectively), and bronchiolar (**F**, *n* = 15, 4, 11, and 6 for mock, 2, 4, and 7 dpi, respectively) epithelia by a blinded investigator. Scale bars: 50 μm. Error bars indicate SEM. Squares indicate males and circles females. *P* < 0.0001 (**B**, **D**, and **E**) and *P* = 0.0047 (**F**) by 1-way ANOVA. *P < 0.05; **P < 0.01; ***P < 0.001; ****P < 0.0001 by Tukey’s post hoc test.

Given prominent viral replication in the airway epithelia and defective MCT in hamster tracheas, we focused on the characterization of airway injury, an area recognized as increasingly important in the consequences of COVID-19 pathogenesis (19–21, 29, 31). Representative images of H&E-stained tracheas (Figure 4A) depict normal pseudostratified and ciliated (apical surface) epithelium in mock hamsters, while ciliated epithelium was highly disrupted and inflamed in SARS-CoV-2 hamsters at 4 dpi. Mononuclear inflammatory cells expanded the submucosa and infiltrated the epithelial mucosal layer. Cilia were largely absent, whereas apoptotic, desquamated epithelial cells that lost the attachment to adjacent epithelia were present. The injury was resolved at 7 dpi with recovered ciliated epithelia, and only occasional submucosal and intraepithelial mononuclear cell infiltration (Figure 4A). Lesions in hamster tracheas were scored according to Gruber et al. (55), and are summarized in Figure 4B. The injury in the upper airways preceded that in the lung, peaked at 2 dpi (5 ± 0, *P* = 0.0038 vs. mock) and 4 dpi (4 ± 1, *P* < 0.0001 vs. mock), and recovered by 7 dpi (2 ± 0, *P* = 0.0184 and 0.0007 vs. 2 and 4 dpi, respectively), consistent with a previous report (54). This trend correlates with the peak at 2 dpi and the subsequent decrease in viral titers in the hamster respiratory system (Figure 1).

To examine the impact on cilia, we labeled unstained tracheal and lung slides for α-tubulin by immunohistochemistry (IHC). We found that the α-tubulin–positive layer along the apical surface of the tracheal epithelia dramatically decreased at 4 dpi compared with mock and then recovered at 7 dpi (Figure 4C). Cilia coverage, the percentage of α-tubulin–positive area along the airway epithelium of tracheas, bronchi, and bronchioles in lungs, was quantified by a blinded pathologist (Figure 4, D–F). Results in the trachea (Figure 4D) demonstrated cilia loss, from a baseline of 90% ± 2% in mock to 40% ± 0% (*P* = 0.0021) and 25% ± 9% (*P* < 0.0001) at 2 and 4 dpi, whereas this was largely restored by 7 dpi (*P* = 0.0055 and *P* < 0.0001 vs. 2 and 4 dpi, respectively), a finding in agreement with tracheal histopathological progression (Figure 4, A and B). This was also consistent with cilia loss in previous histological studies in hamsters (36, 54), including a 95% loss of ciliated area found in hamster tracheas at 4 dpi with a different strain of SARS-CoV-2 (BetaCoV/France/IDF00372/2020) (31). More importantly, the time course of ciliary coverage changes is in parallel with loss of motile CC (Figure 3D) and delayed MCT (Figure 3C) measured by μOCT, indicating that the absence of cilia is the main contributor to the MCT deficit in SARS-CoV-2 infection.

Ciliary loss in the bronchi (Figure 4E) exhibited a progressive fashion similar to that in the trachea, from 98% ± 1% in mock to 52% ± 12% (*P* = 0.0003) and 62% ± 7% (*P* < 0.0001) at 2 and 4 dpi, respectively, with a recovery to 96% ± 2% at 7 dpi (*P* = 0.0052 and 0.0083 vs. 2 and 4 dpi, respectively). However, cilia expression in the smaller airways, i.e., the bronchioles (Figure 4F), remained minimally affected until a slight decrease at 7 dpi (81% ± 6% compared with 96% ± 1% in mock, *P* = 0.0022). The different time patterns can be explained by the steady progression of viral infection from the upper to lower airways with time. Notably, the degree of cilia loss declined from the proximal large airway (trachea) to distal medium (bronchi) and then small airways (bronchioles). This is consistent with the diminished viral distribution along these locations as shown by RNAscope analysis (Figure 1, C and D). Taken together, our results indicate that unlike parenchymal lung injury, direct cytopathic response to viral invasion causes ciliary and airway injury.

### Aberrant ciliary motion and shortened residual cilia.

**Figure 5:**
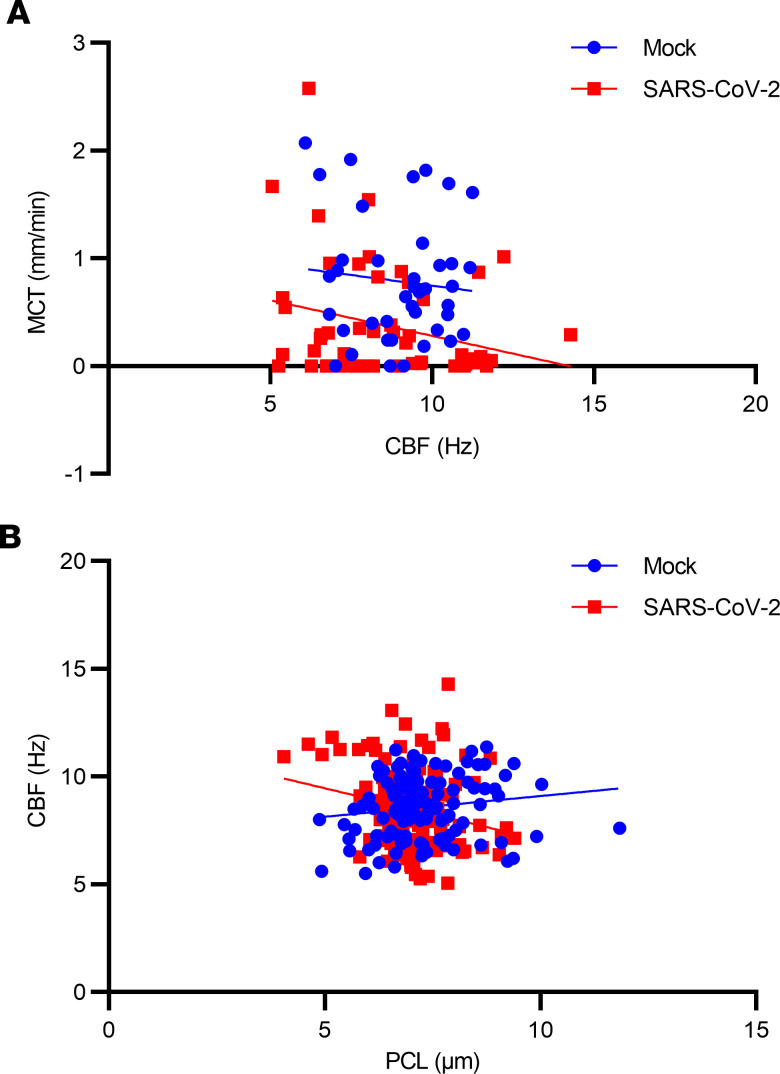
Association of mucociliary clearance functional parameters with delayed MCT in SARS-CoV-2 infection. Values for each region of interest of each hamster trachea were obtained in Figure 3. Linear correlation analysis of MCT with CBF (**A**), and CBF with PCL depth (**B**) in the presence and absence of SARS-CoV-2 infection.

Previous data have established a direct correlation between CBF and MCT in excised human (60), porcine (61), and ferret (62) airways with diseases of mucus stasis such as cystic fibrosis and chronic obstructive pulmonary disease. We assessed whether the relationship between MCT and CBF varied substantially by SARS-CoV-2 infection status using each individual ROI. In mock controls, CBF positively correlated with MCT, whereas CBF negatively correlated with MCT in the presence of SARS-CoV-2 infection (*r* = –0.28, *P* = 0.0390; Figure 5A). A sensitivity analysis examining the correlation of CBF with MCT limited to 4 dpi, when the severity of the functional deficits from SARS-CoV-2 infection was at its peak, also revealed a negative correlation (*r* = –0.41, *P* = 0.0064).

CH has been reported to be reduced in SARS-CoV-2 infection in primary human bronchial epithelial cells as part of the disordered repair associated with ciliated cell infection (31). CBF is affected by the external environment in which they beat; shortened cilia would be expected to encounter less resistance from the overlying mucus gel, and thus potentially greater CBF for the same energy expenditure. This could explain the inverse relationship between CBF and MCT in SARS-CoV-2 infection. Indeed, we observed that PCL depth, a proxy for CH, was inversely associated with CBF, but only when SARS-CoV-2 infection was present (*r* = –0.12, *P* = 0.0122; Figure 5B). Thus, we suspect that ciliary shortening detected at a regional level altered local ciliary beating, partially preserving CBF in these locations.

**Figure 6:**
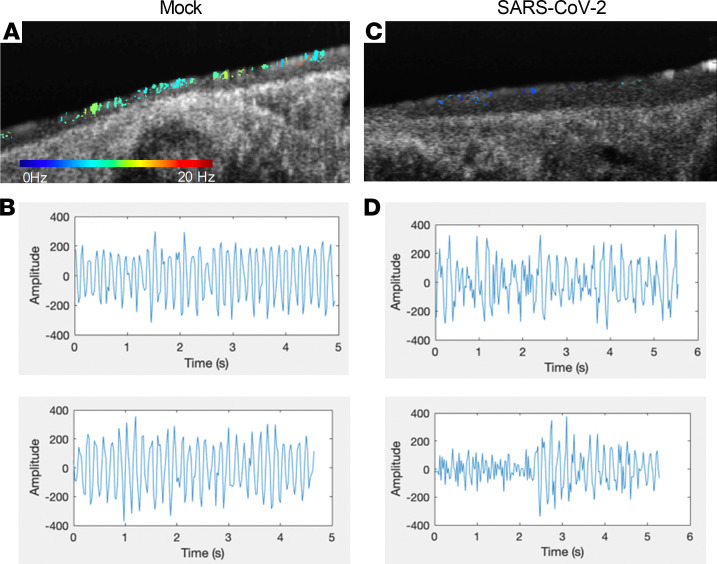
Abnormal ciliary beat frequency map and ciliary motion in SARS-CoV-2–infected hamsters. μOCT images were acquired in Figure 3. Compared with mock controls (**A**), fewer cilia with intact and maintained ciliary beat frequency were evident in SARS-CoV-2–infected hamsters (**C**). Two representative waveform analyses of detected cilia exhibited consistent amplitude and frequency in trachea from mock controls (**B**) compared with erratic amplitude and irregular beat patterns in trachea from SARS-CoV-2–infected hamsters (**D**).

To examine ciliary function further, we examined the waveforms of individual cilia at 4 dpi as compared to mock controls (Figure 6). The representative images of CBF maps showed fewer cilia with intact ciliary motion in SARS-CoV-2–infected hamsters (Figure 6C) as compared with mock controls (Figure 6A). The waveform analysis revealed preserved amplitude and consistent frequency in mock controls (Figure 6B), whereas ciliary waveforms of SARS-CoV-2–infected hamsters exhibited variable amplitude and irregular beat patterns, even when mean CBF was maintained (Figure 6D). Therefore, we conclude that fewer motile cilia combined with altered CH and abnormal ciliary motion of residual cilia contribute to delayed MCT in SARS-CoV-2 infection.

## Discussion

**Figure 7:**
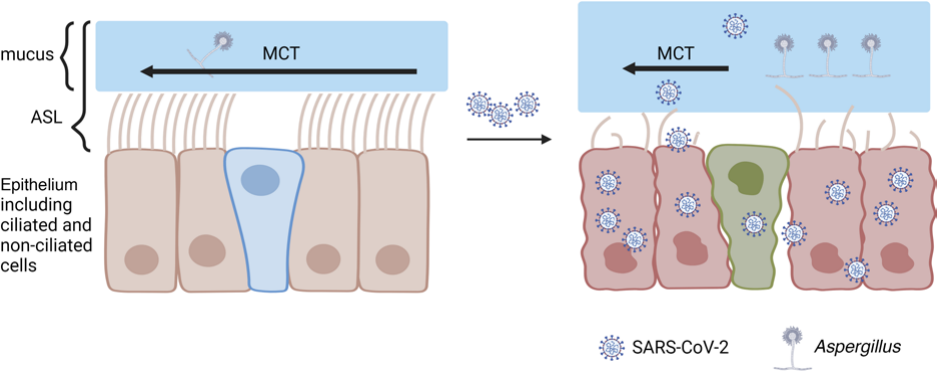
Summary of MCT pathogenesis and disease progression in hamsters. Direct cytopathic effects of viral invasion and replication causes epithelium damage, including motile cilia loss and aberrant ciliary motion of residual cilia that results in MCT deficiency. Thickening of the mucus layer is likely due to the deficit in MCT, although increased mucus production by secretory cells also contributes. Delayed MCT and a thickened mucus layer contribute to viral retention, secondary infections (*Aspergillus*, as an example), and downstream pathogenesis. As viral titer and replication descend from proximal to distal airways over time, cilia loss induced by direct cytopathic effects of viral infection attenuates, whereas pathological injury likely via downstream mediators induced by infection increases. Images created with BioRender.com.

Substantial clinical evidence supports the notion that ciliary function plays an important role in COVID-19 pathogenesis. Here, we quantitatively measured and comprehensively analyzed functional parameters of the MCT apparatus in Syrian hamsters, an animal model of COVID-19 pathogenesis, in parallel with physiological, virological, and pathological analyses of SARS-CoV-2 infection (Figure 7). Noted by markers of viral replication and consequently tissue injury, we demonstrate evidence of steadily descending infection from the large to the medium airways, resulting from direct cytopathic effects of viral infection, and then to small airways and parenchymal lung. SARS-CoV-2 infection caused a 67% decrease in MCT as early as 2 dpi that was sustained through 7 dpi. Results corresponded with virological and pathological evidence of descending infection, primarily involving ciliated epithelial cells of the conducting airways, and noting that recovery of weight loss lagged behind. Corresponding with the functional deficit, we observed the loss of motile cilia, captured by reduced functional ciliary coverage, in addition to abnormal ciliary beating in residual cilia. The pathological analysis confirmed the deficit in ciliated respiratory cells. While we cannot rule out the possibility that secondary postinflammatory events also contributed to cilia loss in the most distal airways, given less evidence of viral titer by RNAscope analysis (Figure 1, C and D) as compared with more proximal airways, the discrepancy between the decreasing degree of cilia loss but increasing downstream injury induced by viral invasion from proximal to distal airways suggests direct effects induced by viral infection on cilia loss in airways. Of importance, some functional deficits in the MCC apparatus persisted through 7 days, as indicated by reduced CC, suggesting a propensity for secondary infection even after viral replication has ceased.

As in prior studies, we used regression analyses of the MCT apparatus to dissect relative contributors to the function decrement. While the cross-sectional analyses showed CBF was largely stable, the correlation analyses of colocalized areas provided additional insight. Results indicated that the reduced ciliary length corresponded to greater CBF, an unexpected finding only observed in the presence of SARS-CoV-2 infection. We suspect that this occurred by reducing ciliary workload consequent to reduced cilia penetration into the mucus layer. Alternatively, it is also possible that CBF was actively responding to the need to accelerate MCT through a feedback loop involving epithelial Ca^2+^ signaling, a mechanism known to occur when cilia encounter high workloads associated with increased mucus viscosity (63, 64). Cilia waveform analysis further suggested deranged ciliary beating and loss of coordinated motion. In total, our findings demonstrate that loss of cilia combined with the functionally inadequate beating of shortened cilia causes severe abnormalities in MCT.

MCT deficiency is likely to have significant clinical consequences. MCT decrements occurred early in the injury process, suggesting it may contribute to the propensity for descending viral infection, whereas parenchymal injury followed in time. The damage of the conducting airways, which occurred early after infection as indicated by histopathology and μOCT, appeared to result from direct cytopathic effects, as evidenced by detection of SARS-CoV-2 by in situ hybridization; in contrast, injury to the alveolar compartment of the lung occurred later in the disease progression and without evidence of SARS-CoV-2, suggesting injury is likely mediated by indirect and downstream proinflammatory pathways. The infection and damage of type II cells potentially is the direct cause of parenchymal damage. The delayed MCT likely has other manifestations critical to COVID-19 pathogenesis. MCT deficiency could contribute to elevated protein levels of mucin in the airways, the detection of mucin mRNA in blood, and abnormal mucus viscosity detected in severe COVID-19 patients (19–21, 65). Mucus accumulation and stasis have been noted in the airways in autopsy studies of COVID-19 (18–23), and has contributed to complications in ICU studies (25–28). In addition, mucus plugs reduce ventilation and alter gas exchange, which is likely to contribute to hypoxia in COVID-19 patients (20). Moreover, the altered function of the MCC apparatus would be expected to encourage secondary bacterial and fungal infections, a relationship that is commonly associated with other diseases of mucus clearance, including postacute influenza (66), and increasingly reported as a late manifestation of COVID-19 (25–28). The persistent functional deficit in motile CC observed in our study, despite the resolution of cilia loss by α-tubulin IHC analysis, suggests its potential to contribute to persistent respiratory manifestations. *Aspergillus* infection is highly associated with abnormal mucus clearance in other diseases (67) and thus may explain its increasingly recognized role as a COVID-19 complication, along with changes in immunity as a response to viral infection (68–71).

The degree of BW loss after infection is comparable to previous hamster models regardless of the differences in the viral strain and inoculation titers (36, 39–41, 43, 44, 46, 48). The preferential viral infection in the respiratory tract (9), the propensity for descending infection, and the specific lesions in the lung have been observed in human autopsy series (40, 56, 57). A 95% loss of ciliated area has been found in hamster tracheas at 4 dpi with a different strain of SARS-CoV-2 (31), consistent with 72% loss of cilia coverage at 4 dpi observed here. Cilia damage after infection has been demonstrated in HTBE cells (29–31) and hamster tracheas (36, 54). The consequences have been detected by decreased speed and nondirectional movement visualized in HTBE cells by polystyrene beads (31). While we focused on the wild-type virus (WA strain) based on timing of the study and necessary validation, it would be interesting to see whether the Omicron (B.1.1.529) variant differs given the increased replication observed in the respiratory tract (72).

Although we did not measure infectious particle titers per se, viral loads by qRT^2^-PCR and RNAscope, particularly when accompanied by sgRNA quantitation and internal consistency between pathology, ciliary function, and viral RNA, could be a reliable and consistent proxy of infectious virus, which has also been indicated in previous reports (36, 41, 47). While our functional evaluation was limited to the trachea, ciliary injury analysis by α-tubulin IHC indicates that MCT decrements are pertinent to the medium and small conducting airways. Downregulation of Foxj1, a regulator of ciliogenesis, has been suggested as the upstream mechanism of cilia loss in SARS-CoV-2–infected HTBE cells immediately after infection (31). The loss of ciliated cells is likely to explain scRNA-seq analysis of BALF from patients with severe COVID-19 showing downregulation of gene transcripts for ciliary structure and function (19).

In summary, we detected and quantitated MCT deficiency in an animal model of COVID-19. By comparing MCT trajectory and time courses of physiological, virological, and pathological progression in hamsters infected with SARS-CoV-2, we suggest that functional deficits of the MCT apparatus predispose to COVID-19 pathogenesis by extending viral retention and is a risk factor for secondary infection. Monitoring abnormal ciliated cell function may indicate disease progression. Adapting the μOCT technology in human upper airways that has been established in our laboratory (60), we are currently evaluating this possibility in human patients. Furthermore, the results strongly suggest that therapies directed toward the MCT apparatus deserve further investigation as a treatment modality.

## Methods

### Study design.

LVG Golden Syrian hamsters were purchased from Charles River (catalog Crl:LVG(SYR)/049) and were inbred in the animal facility at the University of Alabama at Birmingham (UAB). Sex-matched adult hamsters were inoculated intranasally (50 μL per nostril) with 3 × 10^5^ PFU of SARS-CoV-2 (*n* = 26) or vehicle (mock, *n* = 18). Clinical signs, including BW loss, lethargy, fur ruffling, hunched posture, sneezing, coughing, diarrhea, and nasal or ocular discharge were monitored; nasal brushes, oral and rectal swabs were collected; and hamsters were euthanized up to 7 dpi (*n* = 4, 12, and 10 for 2, 4, and 7 dpi, respectively). After euthanasia by intraperitoneal injection of 100 mg/kg pentobarbital, the ventral sides of tracheas were excised and prepared for μOCT imaging followed by fixation in 10% neutral buffered formalin (NBF) (catalog 23-245684, Thermo Fisher Scientific) for histological and IHC analyses; lungs were cleaned and weighed; blood, nasal washes and bronchial alveolar lavage fluid (BALF) from right lung lobes were collected for quantitation of both genomic and viral sgRNA by qRT^2^-PCR; and left lungs were slowly inflated with 3 mL of NBF and then fixed in NBF for histological, IHC, and RNAscope analyses. Tracheas and left lungs were fixed in NBF for 3–5 days to assure viral deactivation before being processed by the UAB Comparative Pathology Laboratory.

### SARS-CoV-2.

The SARS-CoV-2 isolate USA-WA1/2020 was obtained from BEI Resources (catalog NR-52281) and further propagated in Vero E6 cells (catalog C1008, ATCC) through 2 more passages to obtain a working stock of virus at sufficient titer. Viral titers were determined by standard plaque assay (73). Briefly, virus was serially diluted and added to confluent Vero E6 cells grown on 6-well plates. After 1-hour incubation, 1× Minimal Essential Medium (catalog 11430-030, Gibco/Thermo Fisher Scientific) with 2% fetal bovine serum (catalog C788U20, Denville Scientific, Inc.), 1× antibiotic-antimycotics (catalog 15240-062, Gibco/Thermo Fisher Scientific), and 0.6% Avicel CL-611 (FMC BioPolymer) was added, and the infection was allowed to proceed for 72 hours. Plates were fixed by submerging in 10% phosphate-buffered formalin (catalog SF100-20, Thermo Fisher Scientific) for 24 hours, stained with 0.05% neutral red (catalog N2889-100ML, Sigma-Aldrich) for approximately 1 hour, and rinsed in water. Plaques were counted manually from the scanned image of each well. RNA-seq by the UAB Heflin Genomics Core Laboratory indicated fewer than 10 mutations from the parent strain, which is no more than 14% of the sequences annotated. The aliquots of viruses were stored at –80°C for up to 3 months prior to usage. The viral transport medium (VTM) containing Hanks Balanced Salt Solution (catalog 14-025-092, Thermo Fisher Scientific), 2% heat-inactivated fetal bovine serum (catalog 26-140-079, Thermo Fisher Scientific), 100 μg/mL gentamicin (catalog G1397, Sigma-Aldrich), and 0.5 μg/mL amphotericin B (catalog A2942, Sigma-Aldrich) was prepared according to the CDC Standard Operating Procedure (SOP DSR-052-05) (74).

### Viral detection by qRT^2^-PCR.

Nasal brushes were collected using a GUM - 877A Proxabrush (Amazon), and oral and rectal swabs were collected using Medical Packaging Swab-Pak Swabs from Thermo Fisher Scientific (catalog 22-281661). Once collected, tips of brushes and swabs containing samples were placed in 0.5 mL VTM and mixed well. Blood was collected into BD Microtainer Capillary Blood Collectors (catalog 02-675-185, Thermo Fisher Scientific) through cardiac puncture. Serum was isolated within 10 minutes of blood collection to avoid hemolysis and through centrifugation at 1,000*g* for 10 minutes. For nasal washes, the laryngeal or proximal trachea was clamped by hemostats, 0.5 mL VTM was injected into 1 nostril through a blunted needle, and the liquid eluting out of both nostrils was collected and air was then injected to collect the residual liquid in nasal turbinates. BALF was collected from right lung lobes using 2 mL of PBS pH 7.4 (catalog 10-010-023, Thermo Fisher Scientific) with 60% to 80% return.

A Maxwell RSC Viral Total Nucleic Acid Purification Kit (catalog ASB1330, Promega Corporation) was used for RNA isolation. As instructed by the manufacturer, 300 μL of sample was mixed by vortexing for 10 seconds with 330 μL of a freshly made mixture of lysis buffer and proteinase K solution at a 10:1 ratio. Upon incubation at room temperature for 10 minutes and at 56°C for an additional 10 minutes, RNA was extracted utilizing the Maxwell RSC 48 Instrument (Promega Corporation), and qRT^2^-PCR was performed utilizing a QuantStudio 5 Real-Time PCR instrument (Thermo Fisher Scientific). The genomic RNA PCR primers (forward 5′-GACCCCAAAATCAGCGAAAT-3′ and reverse 5′-TCTGGTTACTACTGCCAGTTGAATCTG-3′) and probe (/SFAM/ACCCCGCATTACGTTTGGTGGACC/BHQ_1) targeting the nucleocapsid (N) gene were purchased from Integrated DNA Technologies, Inc. (IDT). AccuPlex SARS-CoV-2 Reference Material (catalog 0505-0126, SeraCare Life Sciences, Inc.) was extracted and amplified in parallel with samples to generate a standard curve enabling viral quantitation. The sg PCR assay utilized a forward primer targeting the leader sequence (5′-CGATCTCTTGTAGATCTGTTCTC-3′, IDT) and the same reverse primer targeting the N gene as for genomic RNA PCR. The sg PCR products were detected by Power SYBR Green RNA-to-C_T_ 1-Step Kit (catalog 4389986, Thermo Fisher Scientific). The sgRNA standard was constructed in-house by amplifying the N gene using primers sgRNA-UF (5′-CCAACCAACTTTCGATCTCTTGTA-3′) and sgRNA-NR1 (5′-AAGGTCTTCCTTGCCATGTTG-3′) from IDT. The amplified band was purified from agarose gel, and this stock standard was quantitated as 2 × 10^13^ copies/mL by NanoDrop One Microvolume UV-Vis Spectrophotometer (Thermo Fisher Scientific), which was serially diluted and utilized directly as the PCR template to generate the standard curve for viral sgRNA quantitation.

### Viral detection by RNAscope.

The viral RNA in the left lung was detected using an RNAscope 2.5 HD Reagent Kit - RED (catalog 322350, Advanced Cell Diagnostics, Inc.) and following the manufacturer’s instructions. Briefly, unstained lung slides were deparaffinized and pretreated with hydrogen peroxide, target retrieval reagent, and then Protease Plus. The viral RNA was hybridized to a probe specific for USA-WA1/2020 strain, V-SARS-CoV-2-N-O1 (catalog 863831, Advanced Cell Diagnostics, Inc.). Following signal amplification, RED solution was added to visualize the signal, and slides were counterstained and then mounted. In parallel, a probe that hybridizes the hamster peptidylprolyl isomerase B gene, Mau-*Ppib* (catalog 890851, Advanced Cell Diagnostics, Inc.), was applied to positive control slides, and a probe for a soil bacterial gene, *DapB* (catalog 320871, Advanced Cell Diagnostics, Inc.), to negative control slides.

### Histological analysis.

H&E-stained trachea and lung slices were blinded and semiquantitatively scored by a board-certified veterinary anatomic pathologist following published criteria (55). Briefly, tracheal, alveolar, and interstitial (AI), bronchiolar and peribronchiolar (BP), and vascular (V) lesions were scored based on subcategories such as necrosis, cell debris, and location-specific criteria with the presence of these lesions = 1 and their absence = 0. The subcategory scores were added up as scores for these sublocations. For the lung, the sum of sublocation scores (AI + BP + V) were multiplied by overall extent of affected area (0–4, with 0 = normal, 1 = 10%–25%, 2 = 25%–50%, 3 = 50%–75%, and 4 >75% of lung surface area displaying pathologic lesion spectra, respectively) of the whole lung slice to generate a final histopathological score of the left lung.

### IHC staining and analysis.

Unstained tracheal and lung slices were labeled for α-tubulin by the UAB Pathology Core Research Laboratory. Standard IHC processes were applied, with antigen retrieval by 0.01 M sodium citrate buffer (pH 6), 1:30,000 dilution of anti–acetylated tubulin (catalog T7451, Sigma-Aldrich), and 1:500 dilution of goat anti–mouse IgG H&L secondary antibody conjugated with HRP (catalog ab6789, Abcam). The percentage of α-tubulin–positive signal (cilia presence) along the apical surface of the tracheal epithelia (cilia coverage) was semiquantitated by a blinded pathologist.

### μOCT.

Excised tracheas from hamsters were imaged as previously described for other species (60–63). Once the larynx area was open and the trachea was exposed under euthanasia, connective tissues around the trachea were removed, the ventral side of the trachea was excised, and immediately placed and maintained on a gauze saturated with room-temperature Dulbecco’s Modified Eagle Medium (catalog 11-965-092, Thermo Fisher Scientific). The trachea was sealed using Parafilm (catalog 50-136-7664, Thermo Fisher Scientific) in a Petri dish with an assembled window made of a scratch- and UV-resistant acrylic sheet of 1/16’’ thickness (catalog 1227T739, McMaster-Carr) to preserve laser transmission and thus imaging quality by μOCT. The instrumentation, methodology, and analysis of μOCT have been previously established (53, 60, 61) and applied to both animal and human tracheas (60–63). Up to 9 ROIs per hamster trachea were imaged. The apical surface of the airway epithelium is covered with a layer of mucus, which is termed the airway surface liquid (ASL) layer. Within the ASL is a thin layer of liquid that covers the cilia and this is termed the periciliary liquid (PCL) layer (Figure 3A). The motility of cilia is characterized by the cilia beat frequency (CBF). Within a frame of view, the proportion of the epithelium covered with motile cilia is determined as motile ciliation coverage (CC). Mucociliary transport rate refers to the speed at which the mucus is transported across the epithelial surface. ASL depth, PCL depth, CBF, mucociliary transport (MCT) rate, and CC on the epithelial surfaces of tracheas were analyzed using ImageJ (NIH) and MATLAB software (MathWorks) (60–63). Due to the dynamic ciliary motion coming in and out of the imaging plane, motile cilia create time-dependent changes in μOCT signal. A Fourier power spectrum was computed for 3 × 3 pixel subregions in the μOCT videos along the motile cilia interface. Low-frequency vibrations were removed by applying a high-pass filter with a cutoff frequency of 3 Hz to negate the confounding signal. The resulting 20 subregions with the highest frequency peak sharpness, defined as the peak power density divided by the total power density, were then analyzed to generate mean CBF for each ROI (53). The frequency at which the peak of the power spectrum occurs indicates the CBF. The CBF map was generated through a MATLAB script to visualize the location and frequency of active cilia across the epithelial cell surface and the proportion of pixels with active cilia quantitated as previously described (75). M-mode (or, motion-mode) refers to the single dimensional view that delineates the path of a structure over time. MCT is calculated by first generating *x*-versus-time graphs in locations containing mucus particles. Reflective mucus particles generate bright and linear tracks in those graphs (indicated by a green arrow in Figure 3B). By determining the angle of the tracks, the lateral displacement of the particles over time, or MCT, was calculated. To characterize ciliary waveforms of detected cilia, the amplitude of the ciliary signal generated by ciliary motion was plotted for selected subregions of interest that were characteristic of mean values (75).

### Statistics.

Data were analyzed using multiple unpaired *t* tests or 1-way or 2-way analysis of variance (ANOVA) followed by recommended Tukey’s or Šídák’s multiple-comparison test, descriptive statistics, and regression methods (both univariable regression and linear mixed models) in Prism 9.2.0 (GraphPad Software, Inc.), SPSS Statistics (IBM), or SAS v9.4. Data are presented as mean ± SEM, with *P* values of less than 0.05 considered statistically significant. Correlations between metrics were calculated using linear or semilog regression methods. A univariable linear regression model was performed to assess the effects of ASL, CH, CC, and CBF on mean MCT. A linear mixed model for repeated measures analysis was used to model MCT as a repeated measure across ROIs (technical and biological replicates), and fixed effect predictors included ROI effects, cohort effects, sex effects, infection effects, and the potential for interaction among them. Random effects included random intercept only. The final models omitted interaction terms between predictor variables and ROI or cohort, as these were not statistically significant and did not alter conclusions.

### Study approval.

All procedures involving animals and SARS-CoV-2 were approved by the Institutional Animal Care and the Use Committee (no. 20232) and Institutional Biosafety Committee (no. 10-262) at the UAB.

## Author contributions

QL, SEP, and SMR designed the study. QL, SEP, SSH, NVH, CMFP, JEPL, JR, JCG, NWH, ERB, TWD, and LTS conducted the research. JCG, LF, HML, JLT, SML, KSH, and GJT provided reagents and techniques. QL, KV, NVH, CMFP, JEPL, JBF, JR, FAD, HK, EO, LY, LJE, and SMR analyzed the data. QL, KV, JBF, JR, NWH, LJE, and SMR wrote the manuscript. SMR supervised the project. All authors had an opportunity to edit the manuscript and approved of its submission.

## Supplementary Material

Supplemental data

Supplemental video 1

Supplemental video 2

## Figures and Tables

**Table 1:**
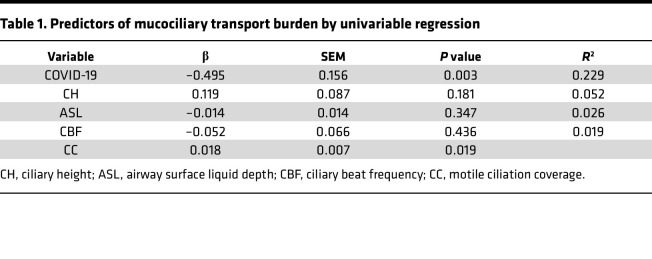
Predictors of mucociliary transport burden by univariable regression

## Version 1 01/10/2023

Electronic publication
