# Supplementary material for: Mucociliary transport deficiency and disease progression in Syrian hamsters with SARS-CoV-2 infection
Source: JCI Insight. 2023 Jan 10;8(1):e163962. doi: 10.1172/jci.insight.163962 (PMC9870055; doi:10.1172/jci.insight.163962)
Supplement: Supplemental data [file jciinsight-8-163962-s179.pdf]

Supplemental Material:

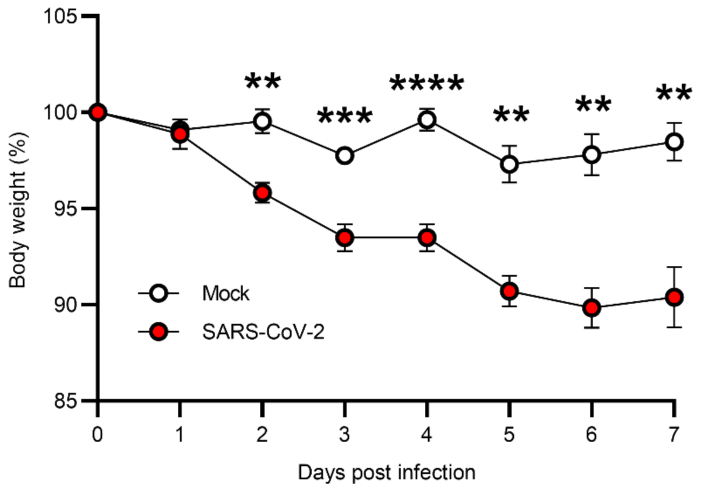

Supplemental Figure 1. Reduced body weight in hamsters through 7 days post-infection with SARS-CoV-2. Golden Syrian hamsters were inoculated intranasally with 3×10^5 plaque-forming units of SARS-CoV-2 or vehicle (mock), body weight was monitored up to 7 days post-infection (dpi; n = 8-25 for infected and n = 4-12 for mock). Error bars = s.e.m. P<0.0001 for time, infection, and interaction by two-way ANOVA; **P<0.01, ***P<0.001 and ****P<0.0001 by Šídák's posthoc test.

Movie S1. Representative real-time recording of mucociliary transport apparatus in tracheas of mock hamsters

Movie S2. Representative real-time recording of mucociliary transport apparatus in hamster tracheas 4 day post SARS-CoV-2 infection
